# Supplementary material for: Differential activation mechanisms of lipid GPCRs by lysophosphatidic acid and sphingosine 1-phosphate
Source: Nat Commun. 2022 Feb 8;13:731. doi: 10.1038/s41467-022-28417-2 (PMC8826421; doi:10.1038/s41467-022-28417-2)
Supplement: Supplementary file 1 — Supplementary Information [file 41467_2022_28417_MOESM1_ESM.pdf]

**Supplementary Information**

# Differential activation mechanisms of lipid GPCRs by lysophosphatidic acid and sphingosine 1-phosphate

Shian Liu, Navid Paknejad, Lan Zhu, Yasuyuki Kihara, Manisha Ray, Jerold Chun, Wei Liu, Richard K. Hite, and Xin-Yun Huang

**Supplementary Table 1**. Cryo-EM data collection, refinement and validation statistics of $S1P_1$ structures.

| | S1P– $S1P_1$–$G\alpha_{i1}\beta_1\gamma_2$ | Siponimod–$S1P_1$–$G\alpha_{i1}\beta_1\gamma_2$ |
|---|---|---|
| **Data collection and processing** | | |
| Magnification | 81000 | 81000 |
| Voltage (kV) | 300 | 300 |
| Electron exposure ($e^-$/Å$^2$) | 24.65 | 23.44 |
| Defocus range (μm) | -0.8 to -1.5 | -0.8 to -1.5 |
| Pixel size (Å) | 1.083 | 1.083 |
| Symmetry imposed | C1 | C1 |
| Initial particle images (no.) | 4,460,634 | 11,076,739 |
| Final particle images (no.) | 449,331 | 1,789,970 |
| Map resolution (Å) | 3.0 | 2.6 |
| FSC threshold | 0.143 | 0.143 |
| Map sharpening *B* factor (Å$^2$) | 122.6 | 116.7 |
| | | |
| **Refinement** | | |
| Initial model used (PDB code) | 3V2Y<br>1GP2 | 3V2Y<br>1GP2 |
| Model resolution (Å) | 3.08 | 2.81 |
| FSC threshold | 0.5 | 0.5 |
| Model composition | | |
| Non-hydrogen atoms | 7035 | 6902 |
| Protein residues | 909 | 894 |
| Ligands | 2 | 2 |
| *B* factors (Å$^2$) | | |
| Protein | 44.66 | 42.35 |
| Ligand | 55.70 | 58.17 |
| R.m.s. deviations | | |
| Bond lengths (Å$^2$) | 0.004 | 0.005 |
| Bond angles (°) | 0.46 | 0.47 |
| Validation | | |
| MolProbity score | 1.57 | 1.57 |
| Clashscore | 2.43 | 3.3 |
| Poor rotamers (%) | 0 | 0 |
| Ramachandran plot | | |
| Favored (%) | 95.31 | 95.11 |
| Allowed (%) | 4.69 | 4.89 |
| Disallowed (%) | 0 | 0 |

**Supplementary Table 2**. Cryo-EM data collection, refinement and validation statistics of $LPA_1$ structures.

| | LPA–$LPA_1$–$G\alpha_{i1}\beta_1\gamma_2$ | LPA–$LPA_1$–$G\alpha_{i1}\beta_1\gamma_2$ (State a) | LPA–$LPA_1$–$G\alpha_{i1}\beta_1\gamma_2$ (State a’) |
|---|---|---|---|
| **Data collection and processing** | | | |
| Magnification | 22,500 | 22,500 | 22,500 |
| Voltage (kV) | 300 | 300 | 300 |
| Electron exposure ($e^-/Å^2$) | 28.2 | 28.2 | 28.2 |
| Defocus range (µm) | -0.8 to -2.2 | -0.8 to -2.2 | -0.8 to -2.2 |
| Pixel size (Å) | 1.064 | 1.064 | 1.064 |
| Symmetry imposed | C1 | C1 | C1 |
| Initial particle images (no.) | 2,235,123 | 1,579,604 | 1,579,604 |
| Final particle images (no.) | 1,588,791 | 31,567 | 32,504 |
| Map resolution (Å) | 2.83 | 3.08 | 3.11 |
| FSC threshold | 0.143 | 0.143 | 0.143 |
| Map sharpening *B* factor ($Å^2$) | -145.7 | -74.9 | -77.8 |
| | | | |
| **Refinement** | | | |
| Initial model used (PDB code) | 4Z34<br>1GP2 | 4Z34<br>1GP2 | 4Z34<br>1GP2 |
| Model resolution (Å) | 2.95 | 3.23 | 3.18 |
| FSC threshold | 0.5 | 0.5 | 0.5 |
| Model composition | | | |
| Non-hydrogen atoms | 7095 | 7151 | 7139 |
| Protein residues | 904 | 907 | 906 |
| Ligands | 1 | 1 | 1 |
| *B* factors ($Å^2$) | | | |
| Protein | 60.82 | 67.82 | 67.36 |
| Ligand | 61.02 | 60.91 | 65.18 |
| R.m.s. deviations | | | |
| Bond lengths ($Å^2$) | 0.002 | 0.002 | 0.002 |
| Bond angles (°) | 0.422 | 0.400 | 0.385 |
| Validation | | | |
| MolProbity score | 1.50 | 1.08 | 0.94 |
| Clashscore | 2.70 | 2.74 | 2.04 |
| Poor rotamers (%) | 0 | 0 | 0 |
| Ramachandran plot | | | |
| Favored (%) | 96.63 | 98.10 | 98.88 |
| Allowed (%) | 3.37 | 1.90 | 1.12 |
| Disallowed (%) | 0 | 0 | 0 |

# S1P$_1$ G$\alpha_i$ $\beta_1\gamma_2$ w/ sphingosine-1-phosphate (S1P)
Cryo-EM Acquisition & Processing

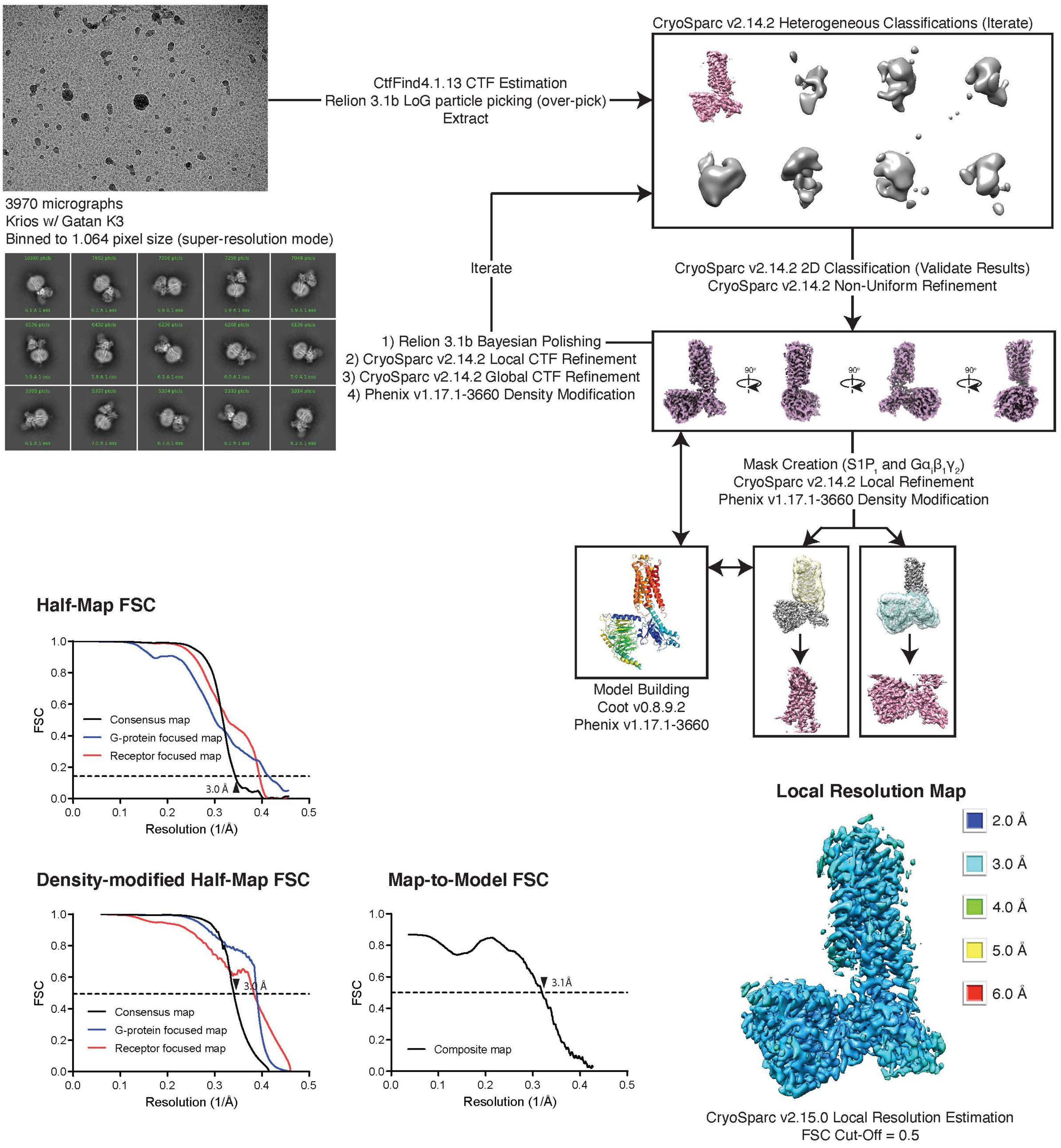


**Supplementary Figure 1. Cryo-EM data acquisition and processing**. In short, 3970 micrographs were collected with low dose and close to focus on a FEI Krios with Gatan K3 direct electron detector. Heterogeneous refinement was used to remove false positive particles, with 2D classification as a form of verification. Once an ideal particle stack was identified, subsequent rounds of heterogeneous refinement were combined with local CTF refinement, Bayesian polishing, and global CTF refinement to further improve the map. Local refinements of S1P1 and Gi helped bring out features in the periphery of the structure. The model was built into the density-modified consensus and local refinements, with the final round of real-space refinement being run against a composite map of the two local refinements generated in Phenix. Overall, most of the model was well represented by density. The alpha-helical domain was less well resolved due to flexibility, and thus not built into the final model.

## S1P$_1$ G$\alpha_i$ $\beta_1\gamma_2$ w/ siponimod
Cryo-EM Acquisition & Processing

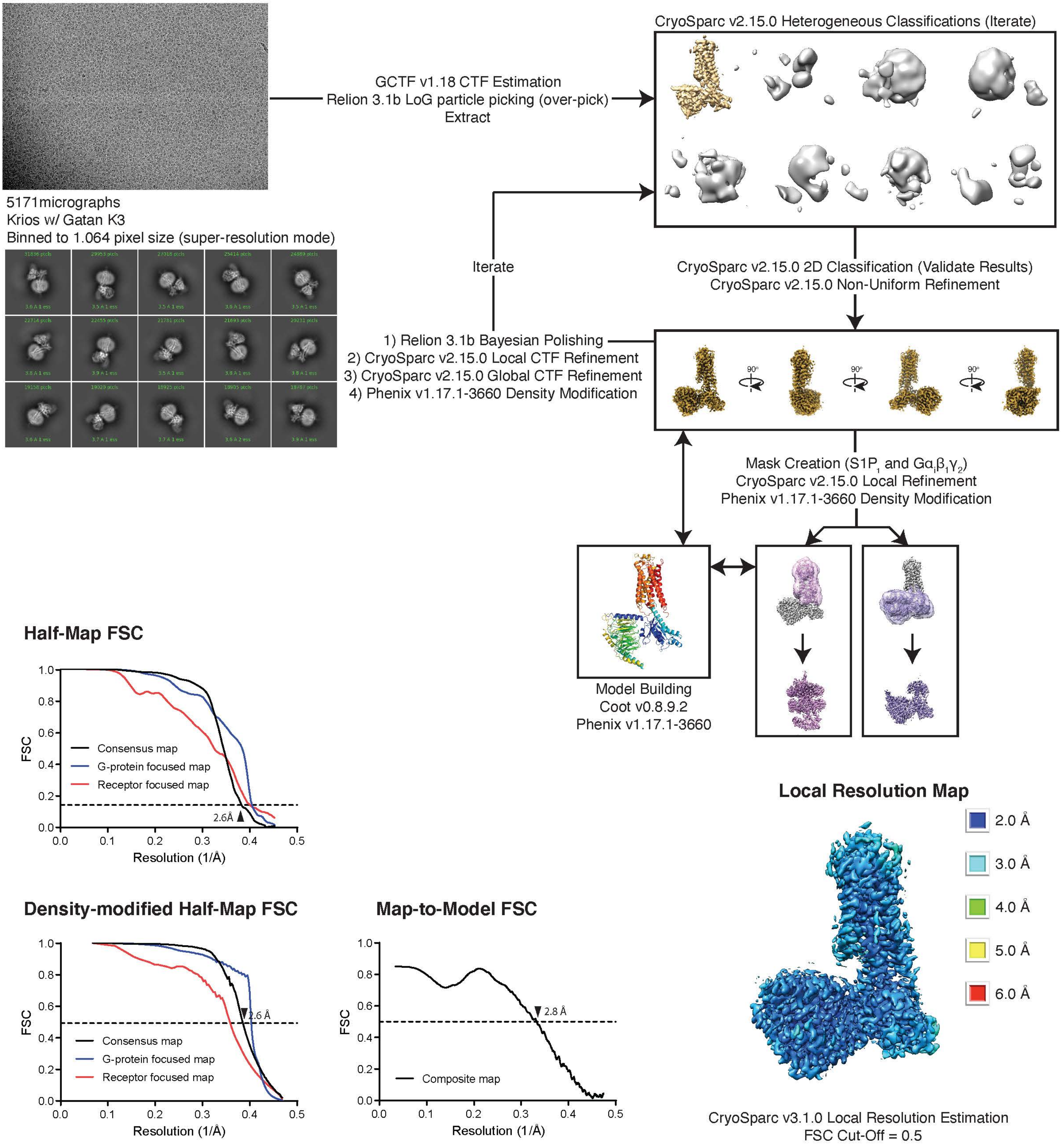


**Supplementary Figure 2. Cryo-EM data acquisition and processing**. In short, 5171 micrographs were collected with low dose and close to focus on a FEI Krios with Gatan K3 direct electron detector. Heterogeneous refinement was used to remove false positive particles, with 2D classification as a form of verification. Once an ideal particle stack was identified, subsequent rounds of heterogeneous refinement were combined with local CTF refinement, Bayesian polishing, and global CTF refinement to further improve the map. Local refinements of S1P1 and Gi helped bring out features in the periphery of the structure. The model was built into the density-modified consensus and local refinements, with the final round of real-space refinement being run against a composite map of the two local refinements generated in Phenix. Overall, most of the model was well represented by density. The alpha-helical domain was less well resolved due to flexibility, and thus not built into the final model.

**LPA$_1$ G$\alpha_i$ $\beta_1\gamma_2$ w/ Lysophosphatidic Acid**
Cryo-EM Acquisition & Processing

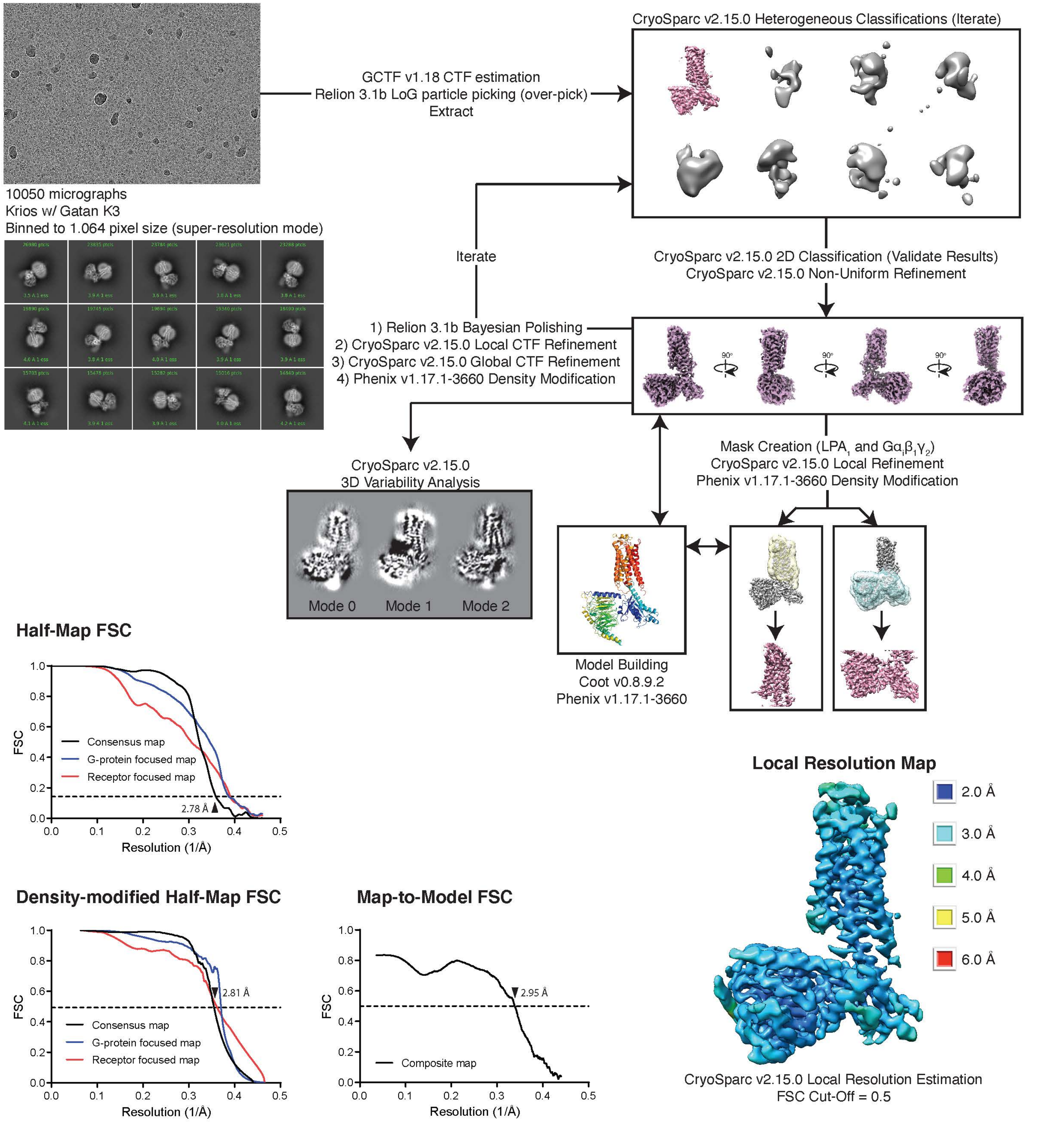


**Supplementary Figure 3: Cryo-EM data processing**. In short, 10050 micrographs were collected with low dose and close to focus on a FEI Krios with Gatan K3 direct electron detector. Heterogeneous refinement was used to remove false positive particles, with 2D classification as a form of verification. Once an ideal particle stack was identified, subsequent rounds of heterogeneous refinement were combined with local CTF refinement, Bayesian polishing, and global CTF refinement to further improve the map. Local refinements of LPA$_1$ and Gi helped bring out features in the periphery of the structure. The model was built into the density-modified consensus and local refinements, with the final round of real-space refinement being run against a composite map of the two local refinements generated in Phenix. Overall, most of the model was well represented by density. The alpha-helical domain was less well resolved due to flexibility, and thus not built into the final model.

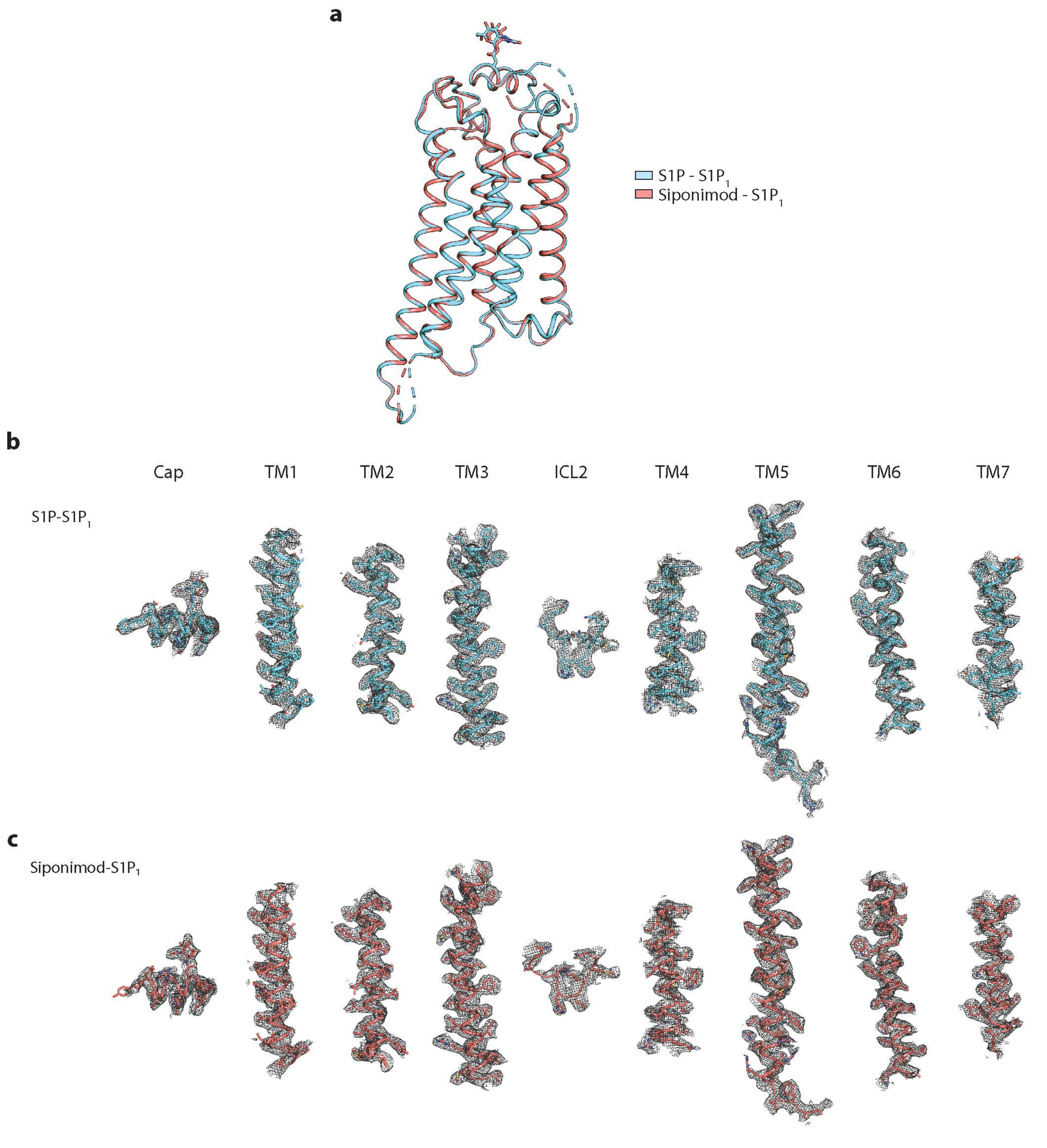


**Supplementary Figure 4**. **Electron microscopy density maps**. **a**, Comparison of the structures of S1P–S1P$_1$ and siponimod –S1P$_1$. **b,c**, Cryo-EM map quality. Representative densities and fitted models are shown for S1P$_1$ in the S1P–S1P$_1$-Gi complex (**b**), and in the siponimod–S1P$_1$-Gi complex (**c**).

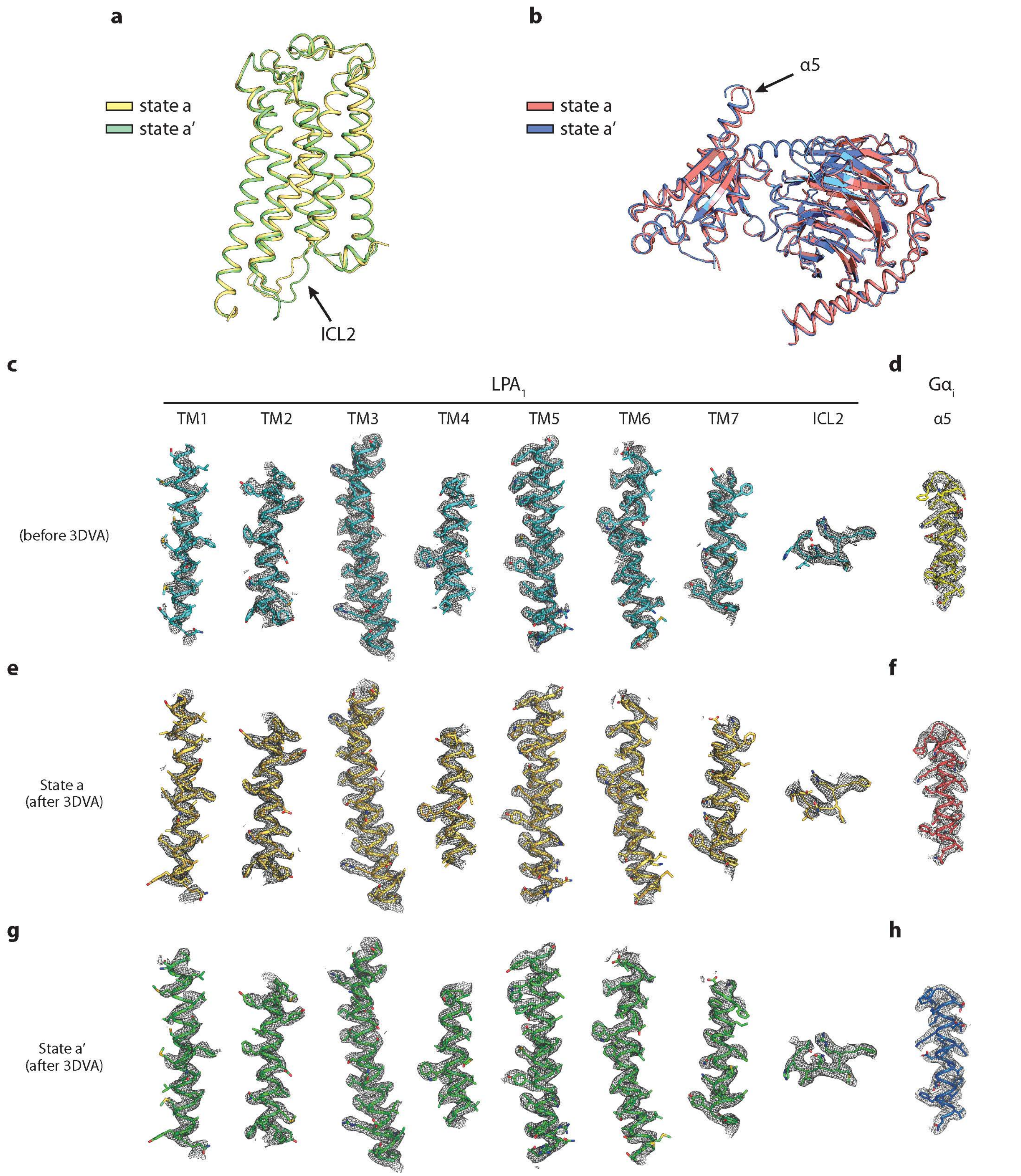


**Supplementary Figure 5**. **Cryo-EM maps**. **a,b**, Comparison of State a and a' from the first component of 3DVA. Superposition of the two structural models aligned for the receptor (**a**) or Gi (**b**). Black arrows indicate the major conformational changes. **c-h**, Cryo-EM map quality. Representative densities and fitted models are shown for $LPA_1$ in the state before 3DVA analysis (**c**), State a after 3DVA (**e**), and State a' after 3DVA (**g**), as well as the $\alpha$5-helices of $G\alpha_i$ (**d, f, h**) in the three states.

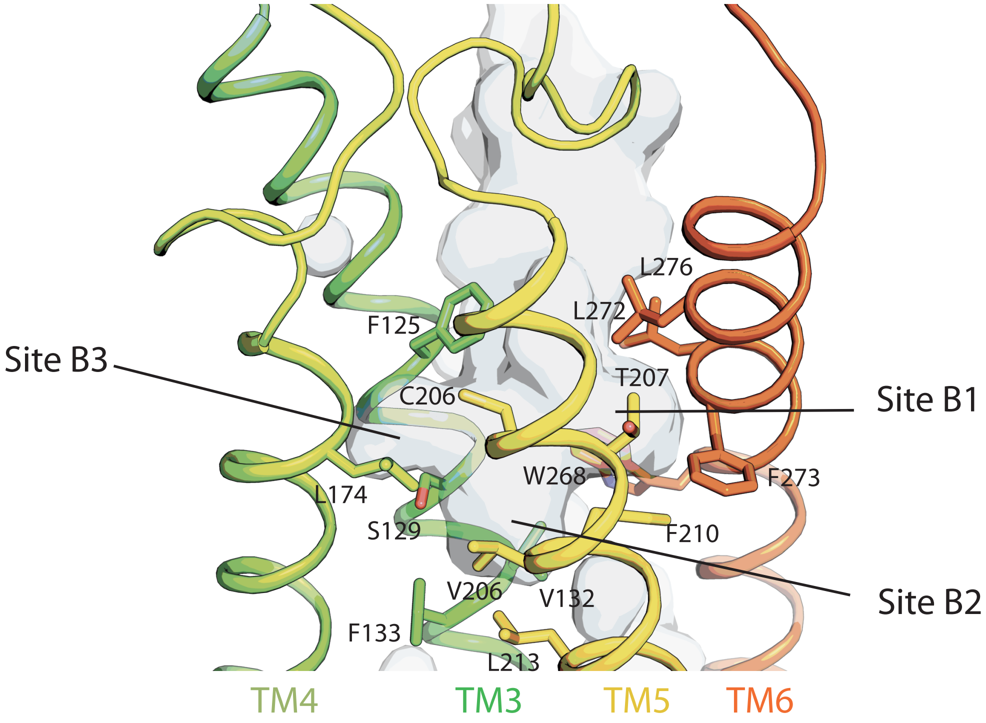


**Supplementary Figure 6. Residues in the side binding pockets B1, B2 and B3 in $S1P_1$.** The cartoon structure of $S1P_1$ is overalyed with the slice through view of the ligand binding pockets of $S1P_1$ with the side binding pockets B1, B2 and B3 indicated. The sidechains of the residues in B1, B2 and B3 are shown.

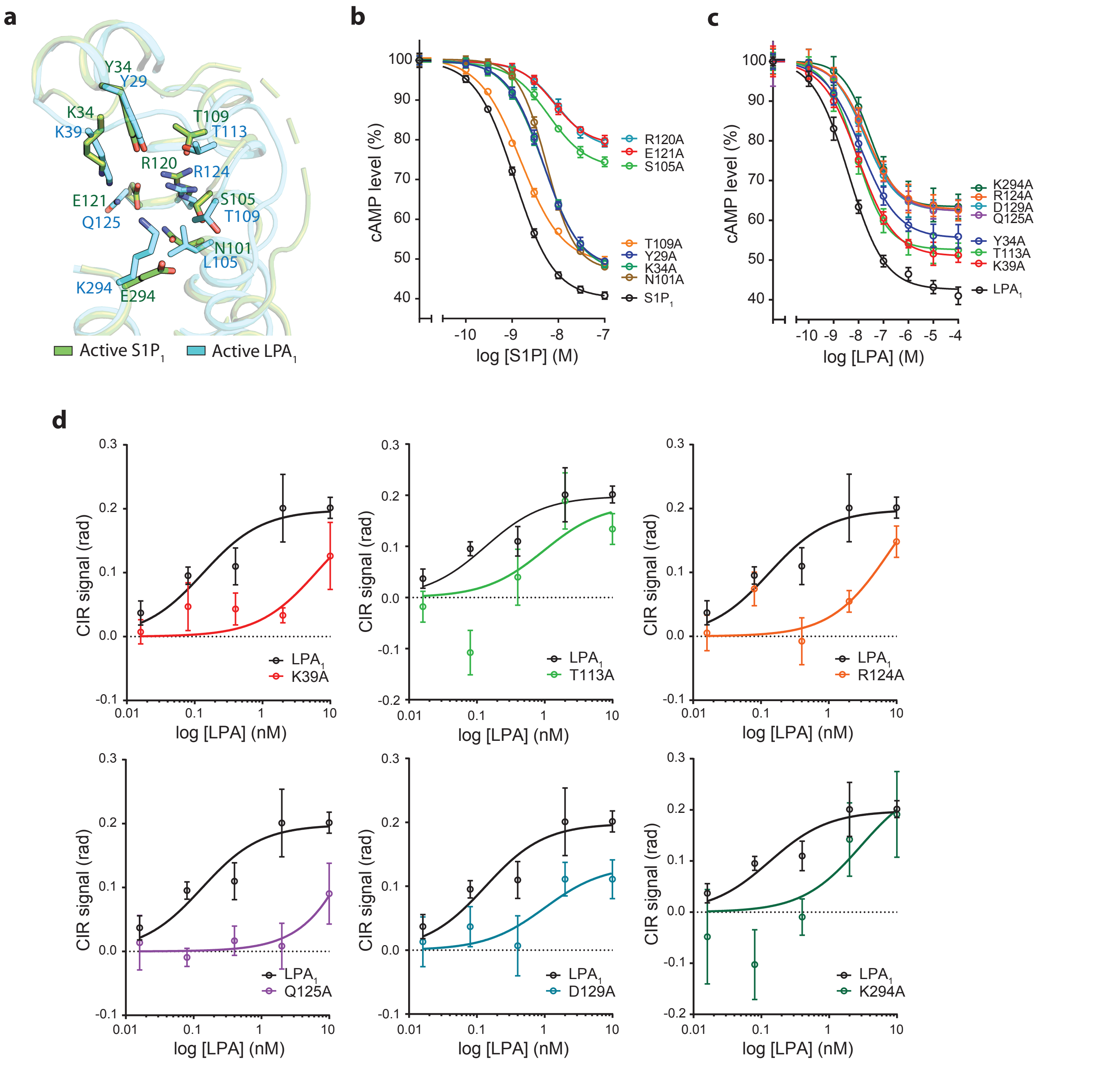


**Supplementary Figure 7. Functional studies of residues in $S1P_1$ and $LPA_1$.** (**a**) Comparison of the active $S1P_1$ and the active $LPA_1$ structures with critical residues shown. (**b**) cAMP assays of wild-type and mutant $S1P_1$ in CHO cells. (**c**) cAMP assays of wild-type and mutant $LPA_1$ in B103 cells. (**d**) Ligand-binding assays of wild-type and mutant $LPA_1$ in B103 cells. Wild-type (WT, in black color), and various mutants of $LPA_1$ were stably expressed in B103 cells. A free solution assay, where the receptor ($LPA_1$/mutants) containing nanovesicles and unlabeled 18:1 LPA ligand were freely moving into solution, was used in a native environment of the binding partners (18:1 LPA-$LPA_1$) (see the Methods). Individual curves from the mutants were comapred with the same curve from the wild-type receptor. Data are shown as mean ± SD. n = 3 independent experiments. Source data are provided as a Source Data file.

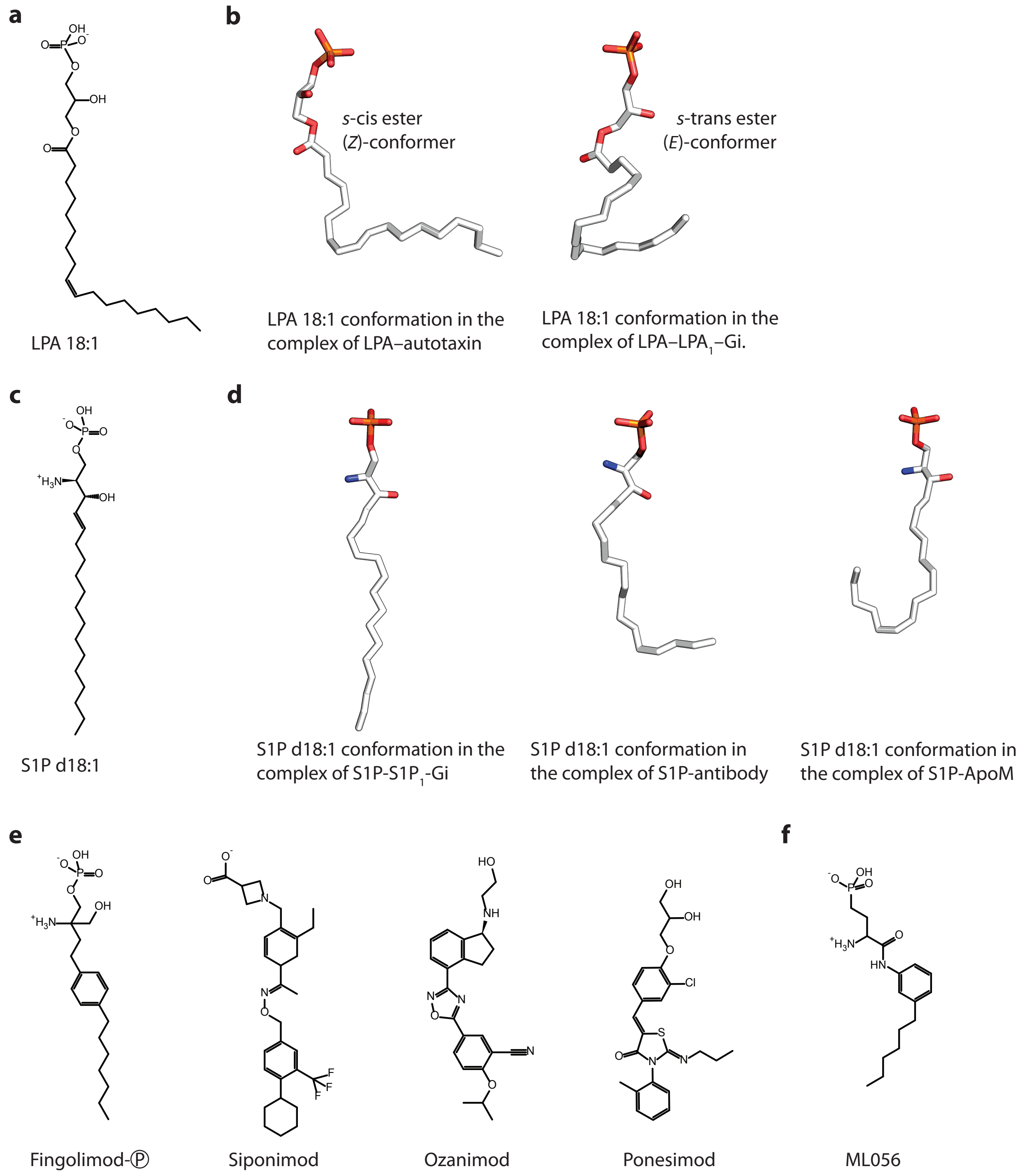


**Supplementary Figure 8. Chemical conformations of ligands for LPA and S1P receptors**. (***a***) Chemical structure of LPA 18:1. (**b**) Structures of LPA 18:1 in the complex of LPA–autotaxin or of LPA–$LPA_1$–Gi. (**c**) Chemical structure of S1P d18:1. (**d**). Structures of S1P d18:1 in the complex of S1P–$S1P_1$–Gi, S1P–antibody, or S1P–ApoM. (**e**) Chemical structures of S1P receptor-targeting MS-treatment drugs. (**f**) Chemical structure of the $S1P_1$ antagonist ML056.

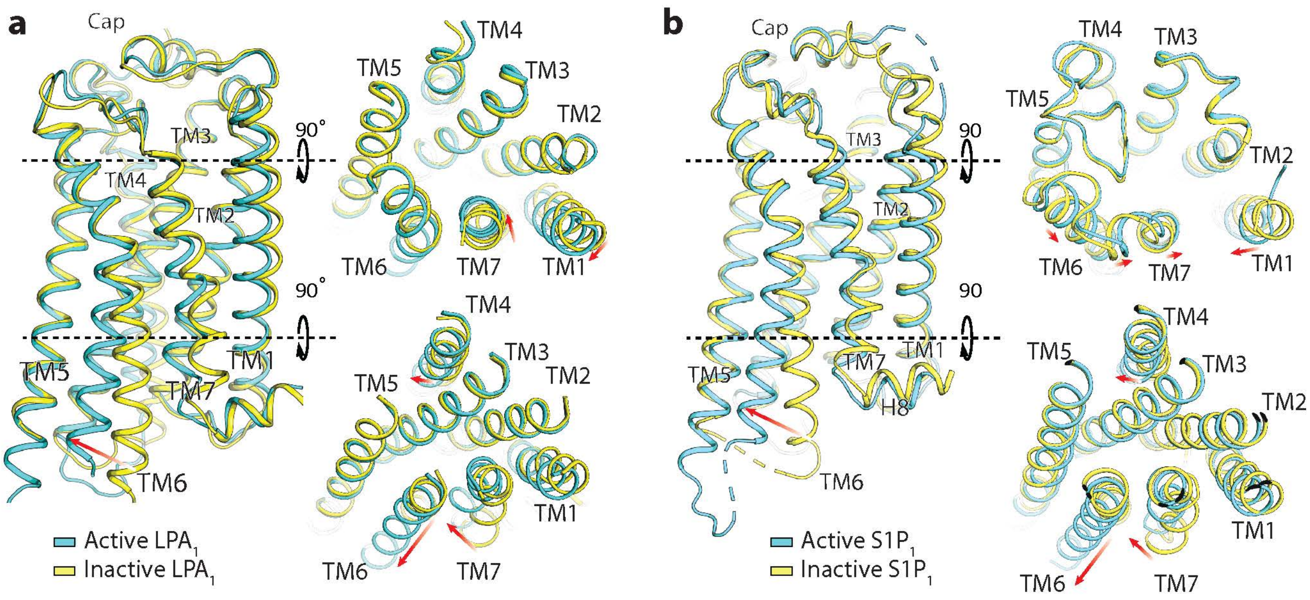


**Supplementary Figure 9. Structural comparisons of inactive and active $LPA_1$ and $S1P_1$. a,** Structural comparison of the inactive (PDB 4Z34) and active (current study) states of $LPA_1$. **b,** Structural comparison of the inactive (PDB 3V2Y) and active (current study) states of $S1P_1$.

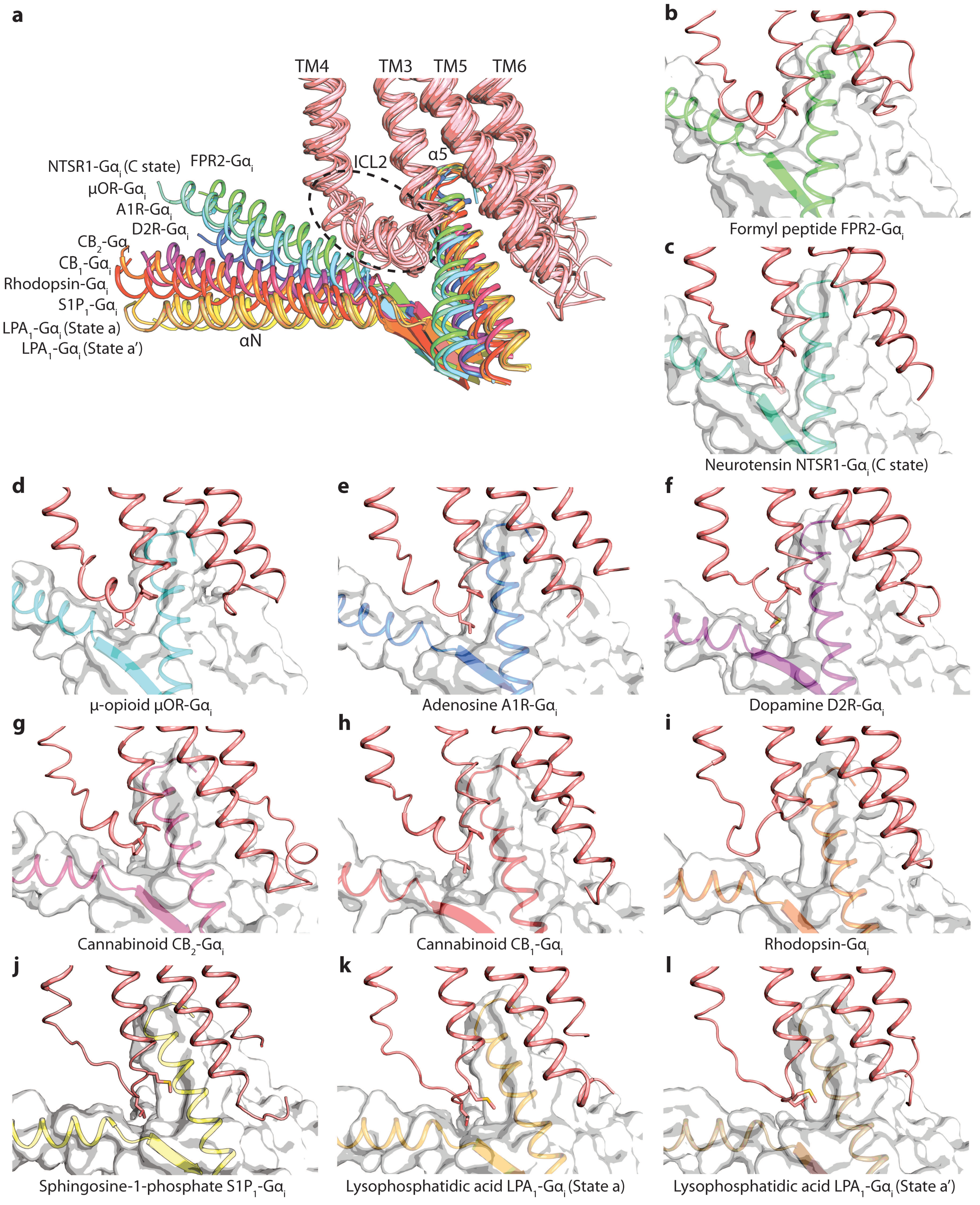


**Supplementary Figure 10. Comparisons of the interactions between ICL2 of family A GPCRs and G$\alpha_i$.** An ensemble (**a**) and individual (**b-l**) displays are presented to show G$\alpha_i$ interactions with ICL2 of FPR2 (PDB: 6omm), Neurotensin (C state) (PDB: 6os9), μOR (PDB: 6dde), A1R (PDB: 6d9h), D2R (PDB: 6vms), $CB_2$ (PDB: 6pt0), $CB_1$ (PDB: 6n4b), Rhodopsin (PDB: 6cmo), $S1P_1$ (this work), $LPA_1$ (State a) (this work) and $LPA_1$ (State a') (this work).

**LPA$_1$ G$\alpha_i$ $\beta_1\gamma_2$ w/ Lysophosphatidic Acid**

Processing of the cluster analysis-isolated states

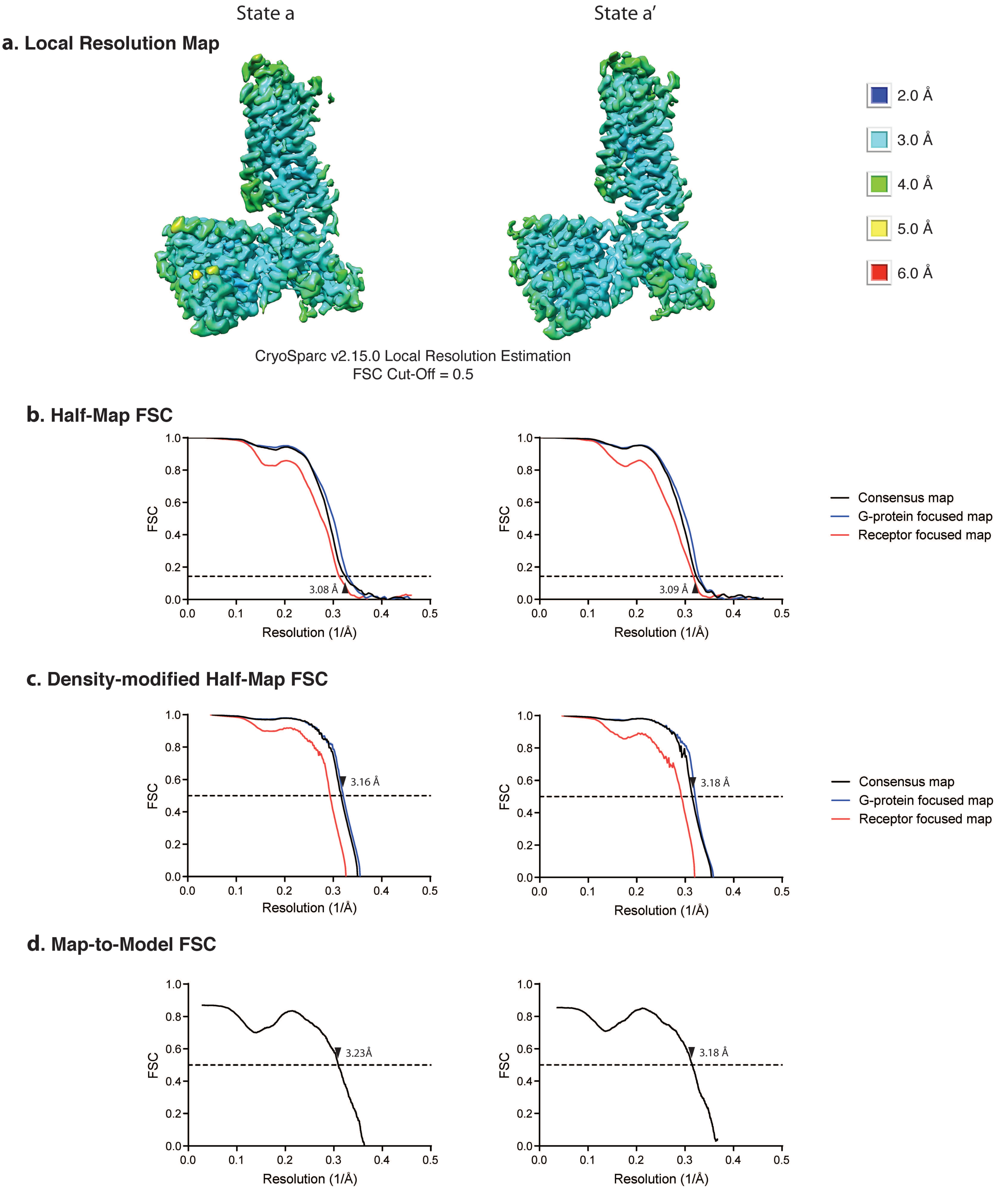


**Supplementary Figure 11. 3D variability analysis (3DVA) in CryoSparc v2 was used to cluster particles based on principal components of movement.** Manual inspection of the maps generated from 20 clusters led to the identification of two farthest positionings for the G-protein relative to the GPCR. Clusters that fit either position were grouped, and our standard processing workflow was used to generate density modified composite maps for each state (see Materials & Methods for details). (**a**) Local resolution maps for both states. (**b**) Half-map FSC curves for the consensus, G-protein focused, and GPCR focused refinements for both states. (**c**) Half-map FSC curves after applying density modification to each refinement for both states. (**d**) Map-to-model FSC curves for the final models refined against a composite map of the three density-modified refinements.

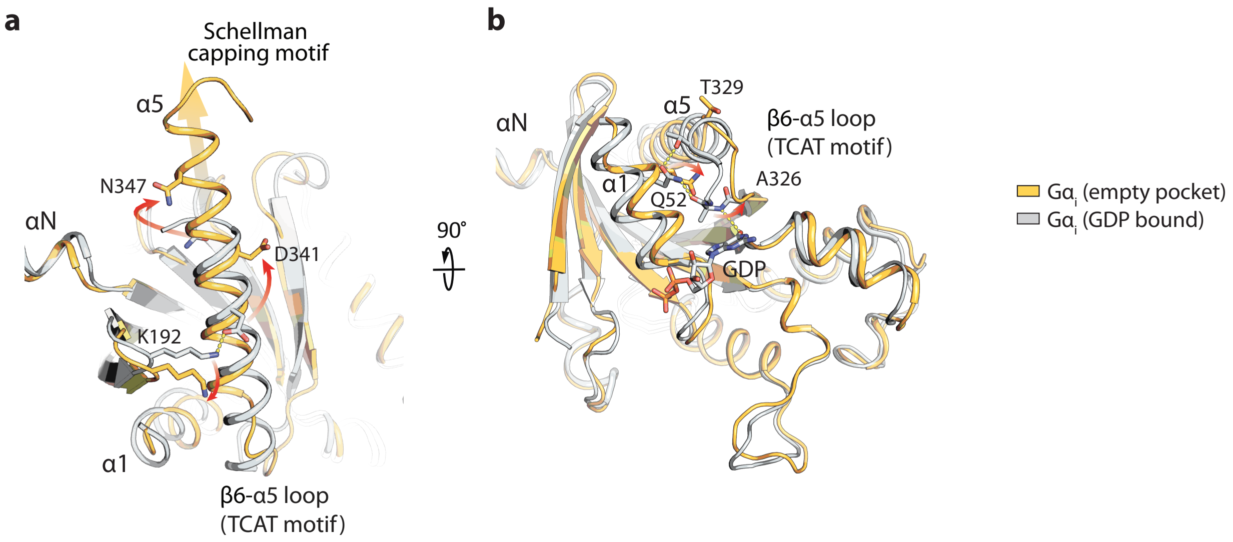


**Supplementary Figure 12. Structural basis of the activation of Gi by LPA-bound $LPA_1$.** (**a)** Disruptions of intra-molecular interactions of G**α**$_i$ during Gi activation by LPA-bound $LPA_1$. An ionic interaction between the sidechain of D341 in the **α**5-helix and the sidechain of K192 in the β2-β3 loop in the inactive G**α**$_i$(GDP)Gβγ trimer (in gray) is broken. D341 forms new interactions with K345 and E318 in the complex of LPA–$LPA_1$–Gi (in light orange). (**b)** An interacting network involving the sidechain of Q52 in the **α**1-helix, the backbone carbonyl of A326 in the β6-**α**5 loop, and the sidechain of T329 in the **α**5-helix is disrupted.

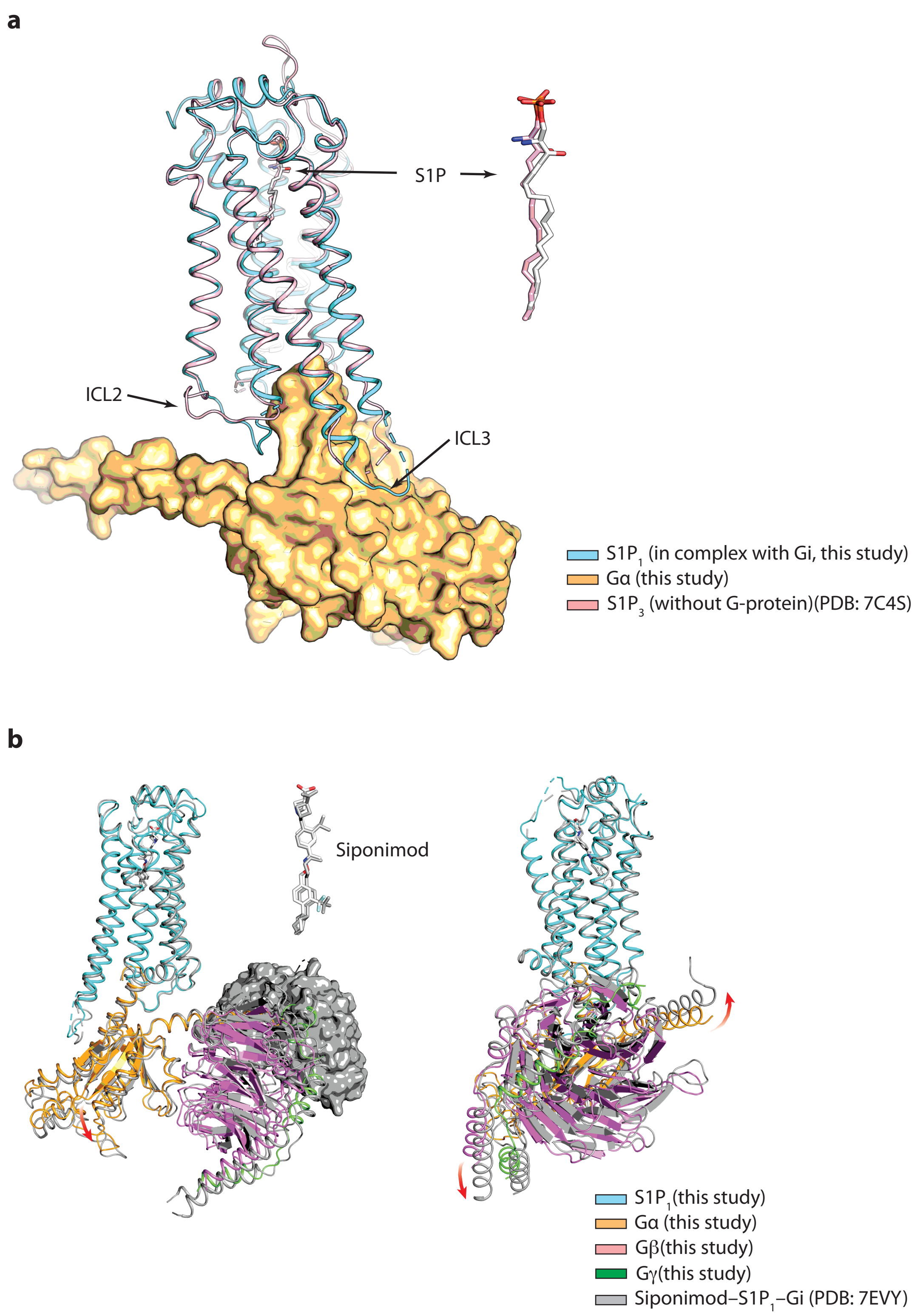


**Supplementary Figure 13. Structural comparisons. a,** Comparison of the complex of S1P–S1P$_1$-Gi and S1P–S1P$_3$ (without G-protein). S1P$_1$ and S1P$_3$ have similar S1P-binding pockets with similar S1P conformation. **b,** Comparison of the complex of Siponimod–S1P$_1$-Gi from the current study and the one recently published (PDB 7EVY). While the conformations of S1P1 and Siponimod are similar, the positions of Gi-proteins are slightly different.
