# Supplementary material for: Differential activation mechanisms of lipid GPCRs by lysophosphatidic acid and sphingosine 1-phosphate
Source: Nat Commun. 2022 Feb 8;13:731. doi: 10.1038/s41467-022-28417-2 (PMC8826421; doi:10.1038/s41467-022-28417-2)
Supplement: Supplementary file 3 — Description of Additional Supplementary Files [file 41467_2022_28417_MOESM3_ESM.pdf]

Description of Additional Supplementary Files

File name: Supplementary Movie 1

Description: 3DVA motion mode 1 of the LPA-bound LPA1 and Gi complex.

File name: Supplementary Movie 2

Description: 3DVA motion mode 2 of the LPA-bound LPA1 and Gi complex.

File name: Supplementary Movie 3

Description: 3DVA motion mode 3 of the LPA-bound LPA1 and Gi complex.

File name: Supplementary Movie 4

Description: 3DVA motion mode 4 of the LPA-bound LPA1 and Gi complex.

File name: Supplementary Movie 5

Description: 3DVA motion mode 5 of the LPA-bound LPA1 and Gi complex.

File name: Supplementary Movie 6

Description: 3DVA motion mode 6 of the LPA-bound LPA1 and Gi complex.

File name: Supplementary Movie 7

Description: 3DVA motion mode 1 of the S1P-bound S1P1 and Gi complex.

File name: Supplementary Movie 8

Description: 3DVA motion mode 2 of the S1P-bound S1P1 and Gi complex.

File name: Supplementary Movie 9

Description: 3DVA motion mode 3 of the S1P-bound S1P1 and Gi complex.

File name: Supplementary Movie 10

Description: 3DVA motion mode 4 of the S1P-bound S1P1 and Gi complex.

File name: Supplementary Movie 11

Description: 3DVA motion mode 5 of the S1P-bound S1P1 and Gi complex.

File name: Supplementary Movie 12

Description: 3DVA motion mode 6 of the S1P-bound S1P1 and Gi complex.

File name: Supplementary Movie 13

Description: 3DVA motion mode 1 of the Siponimod-bound S1P1 and Gi complex.

File name: Supplementary Movie 14

Description: 3DVA motion mode 2 of the Siponimod-bound S1P1 and Gi complex.

File name: Supplementary Movie 15

Description: 3DVA motion mode 3 of the Siponimod-bound S1P1 and Gi complex.

File name: Supplementary Movie 16

Description: 3DVA motion mode 4 of the Siponimod-bound S1P1 and Gi complex.

File name: Supplementary Movie 17

Description: 3DVA motion mode 5 of the Siponimod-bound S1P1 and Gi complex.

File name: Supplementary Movie 18

Description: 3DVA motion mode 6 of the Siponimod-bound S1P1 and Gi complex.
