# Supplementary material for: Differential activation mechanisms of lipid GPCRs by lysophosphatidic acid and sphingosine 1-phosphate
Source: Nat Commun. 2022 Feb 8;13:731. doi: 10.1038/s41467-022-28417-2 (PMC8826421; doi:10.1038/s41467-022-28417-2)
Supplement: Supplementary file 22 — Reporting Summary [file 41467_2022_28417_MOESM22_ESM.pdf]

nature portfolio

Corresponding author(s): Xin-Yun Huang

Last updated by author(s): Dec. 30, 2021

Reporting Summary

Nature Portfolio wishes to improve the reproducibility of the work that we publish. This form provides structure for consistency and transparency in reporting. For further information on Nature Portfolio policies, see our Editorial Policies and the Editorial Policy Checklist.

Statistics

For all statistical analyses, confirm that the following items are present in the figure legend, table legend, main text, or Methods section.

n/a	Confirmed	
☐	☒	The exact sample size (*n*) for each experimental group/condition, given as a discrete number and unit of measurement
☐	☒	A statement on whether measurements were taken from distinct samples or whether the same sample was measured repeatedly
☒	☐	The statistical test(s) used AND whether they are one- or two-sided<br>*Only common tests should be described solely by name; describe more complex techniques in the Methods section.*
☒	☐	A description of all covariates tested
☒	☐	A description of any assumptions or corrections, such as tests of normality and adjustment for multiple comparisons
☐	☒	A full description of the statistical parameters including central tendency (e.g. means) or other basic estimates (e.g. regression coefficient) AND variation (e.g. standard deviation) or associated estimates of uncertainty (e.g. confidence intervals)
☒	☐	For null hypothesis testing, the test statistic (e.g. *F*, *t*, *r*) with confidence intervals, effect sizes, degrees of freedom and *P* value noted<br>*Give P values as exact values whenever suitable.*
☒	☐	For Bayesian analysis, information on the choice of priors and Markov chain Monte Carlo settings
☒	☐	For hierarchical and complex designs, identification of the appropriate level for tests and full reporting of outcomes
☒	☐	Estimates of effect sizes (e.g. Cohen's *d*, Pearson's *r*), indicating how they were calculated

Our web collection on statistics for biologists contains articles on many of the points above.

Software and code

Policy information about availability of computer code

Data collection	SerialEM 3.7 and Leginon 3.5
Data analysis	MotionCorr2 v1.2.1, CTFfind v4.1.10, Relion 3.0 v3.0, CryoSparc v2.14.2 and 2.15.0, coot v0.8.92, Phenix v1.17.1-3660, Chimera v1.14, Pymol 2.5 and GraphPad Prism 8.

For manuscripts utilizing custom algorithms or software that are central to the research but not yet described in published literature, software must be made available to editors and reviewers. We strongly encourage code deposition in a community repository (e.g. GitHub). See the Nature Portfolio guidelines for submitting code & software for further information.

Data

Policy information about availability of data

All manuscripts must include a data availability statement. This statement should provide the following information, where applicable:

- Accession codes, unique identifiers, or web links for publicly available datasets
- A description of any restrictions on data availability
- For clinical datasets or third party data, please ensure that the statement adheres to our policy

The cryo-EM reconstructions of the LPA1-Gi-LPA, LPA1-Gi-LPA (state a), LPA1-Gi-LPA (state a'), S1P1-Gi-S1P and S1P1-Gi-Siponimod complexes have been deposited in the Electron Microscopy Data Bank (EMDB) under ID codes EMD-25819, EMD-25820, EMD-25821, EMD-25822, and EMD-25823, respectively. The corresponding atomic models have been deposited in the Protein Data Bank (PDB) under ID codes 7TD0, 7TD1, 7TD2, 7TD3, and 7TD4, respectively.

Field-specific reporting

Please select the one below that is the best fit for your research. If you are not sure, read the appropriate sections before making your selection.

☒ Life sciences ☐ Behavioural & social sciences ☐ Ecological, evolutionary & environmental sciences

For a reference copy of the document with all sections, see nature.com/documents/nr-reporting-summary-flat.pdf

Life sciences study design

All studies must disclose on these points even when the disclosure is negative.

Sample size	No sample size calculations were performed. For cryo-EM samples, eight grids of each sample were pre-screened to identify the optimal grid for data collection. The number of grids screened were random and was not limited by any experimental parameter. Biochemical experiments were repeated three times.
Data exclusions	No data were excluded from the analyses.
Replication	All the cAMP assays and interferometry assays were repeated at least three times, and the data are represented as mean ± SD of the three independent experiments. All attempts at replication were successful for cAMP and interferometry assays.
Randomization	Samples were not allocated to groups. All cryo-EM particles used for structure determination adopt random orientations in ice on the grid. Division of particles into random halves is automatically performed using 3D reconstruction by Relion 3.0 and CryoSparc v2.14.2 and 2.15.0.
Blinding	Blinding is not applicable for this study, as group allocation is not used.

Reporting for specific materials, systems and methods

We require information from authors about some types of materials, experimental systems and methods used in many studies. Here, indicate whether each material, system or method listed is relevant to your study. If you are not sure if a list item applies to your research, read the appropriate section before selecting a response.

Materials & experimental systems

n/a	Involved in the study	
☒	☐	Antibodies
☐	☒	Eukaryotic cell lines
☒	☐	Palaeontology and archaeology
☒	☐	Animals and other organisms
☒	☐	Human research participants
☒	☐	Clinical data
☒	☐	Dual use research of concern

Methods

n/a	Involved in the study	
☒	☐	ChIP-seq
☒	☐	Flow cytometry
☒	☐	MRI-based neuroimaging

Eukaryotic cell lines

Policy information about cell lines

Cell line source(s)	Sf9 and CHO cells were purchased from ATCC. B103 cells were previously established in our collaborator Dr. Jerold Chun's lab.
Authentication	Sf9 cells and CHO cells were authenticated by morphology at ATCC . B103 cells were authenticated by morphology by our collaborator Dr. Jerold Chun's lab.
Mycoplasma contamination	All cell lines were tested negative for mycoplasma contamination.
Commonly misidentified lines (See ICLAC register)	No commonly misidentified cell lines were used in this study.
